# The Three Glories

**Published:** 1857-11

**Authors:** 


### THE THREE GLORIES; OR, TWO TO ONE.

OF our merchant princes, there are two classes, since the financial tornado has swept across the commercial world. Those who withstood its fury walk now with a firmer tread and higher beating hearts; their glory is, that they still stand. Of the fallen, there are those who fell, proud as ever to-day, because they fell honorably. But our wives and daughters revel in a glory that towers a head and shoulders above all others, and throws all others into the shade—the glory of purchasing dry goods at half price.

---

### TRAPS.

MOSQUITO traps, flies, roaches, &c., are furnished by J. S. Clough, 168 Broadway—certain death to all who enter them.

Boy trap, a girl in her teens; man trap, a taking young widow. Bug trap, flies, insects, and the like: take a pound and a half of common rosin, and a pint of sweet oil, place them in a vessel over the fire, until the rosin is melted, stir them well and when cool it forms a liquid; put it on any piece of cloth, linen, or other flexible material, with a brush, and then wrap it around the stem or body. It remains sticky and clammy for a long time. *Piesse* calls this REZOIL; and says, that birds, cats, and mice give it a wide berth.

*Invalid Trap.* Let him know that you have something which cured somebody else, worse than he, but that you will not tell its name. He will pay you well for it—the higher the price, the more anxious will he be to get hold of it. But tell him that it is a glass of spring water, to be had by walking up a steep mountain five times a day, and he will have nothing to do with it.

